# Towards discovery: an end-to-end system for uncovering novel biomedical relations

**DOI:** 10.1093/database/baae057

**Published:** 2024-07-11

**Authors:** Tiago Almeida, Richard A A Jonker, Rui Antunes, João R Almeida, Sérgio Matos

**Affiliations:** IEETA/DETI, LASI, University of Aveiro, Campus Universitário de Santiago, Aveiro 3810-193, Portugal; IEETA/DETI, LASI, University of Aveiro, Campus Universitário de Santiago, Aveiro 3810-193, Portugal; IEETA/DETI, LASI, University of Aveiro, Campus Universitário de Santiago, Aveiro 3810-193, Portugal; IEETA/DETI, LASI, University of Aveiro, Campus Universitário de Santiago, Aveiro 3810-193, Portugal; IEETA/DETI, LASI, University of Aveiro, Campus Universitário de Santiago, Aveiro 3810-193, Portugal

## Abstract

Biomedical relation extraction is an ongoing challenge within the natural language processing community. Its application is important for understanding scientific biomedical literature, with many use cases, such as drug discovery, precision medicine, disease diagnosis, treatment optimization and biomedical knowledge graph construction. Therefore, the development of a tool capable of effectively addressing this task holds the potential to improve knowledge discovery by automating the extraction of relations from research manuscripts. The first track in the BioCreative VIII competition extended the scope of this challenge by introducing the detection of novel relations within the literature. This paper describes that our participation system initially focused on jointly extracting and classifying novel relations between biomedical entities. We then describe our subsequent advancement to an end-to-end model. Specifically, we enhanced our initial system by incorporating it into a cascading pipeline that includes a tagger and linker module. This integration enables the comprehensive extraction of relations and classification of their novelty directly from raw text. Our experiments yielded promising results, and our tagger module managed to attain state-of-the-art named entity recognition performance, with a micro F1-score of 90.24, while our end-to-end system achieved a competitive novelty F1-score of 24.59. The code to run our system is publicly available at https://github.com/ieeta-pt/BioNExt.

**Database URL**: https://github.com/ieeta-pt/BioNExt

## Introduction

Biomedical relation extraction is essential for understanding the vast and ever-growing body of biomedical literature. By identifying connections between diseases, drugs, genes and sequence variants, we can enhance clinical decision-making through detailed drug–disease interactions. Besides, this also accelerates the discovery of new drug targets, keeps knowledge bases current with the latest research and streamlines the retrieval of biomedical information (31).

Historically, the majority of datasets used for biomedical relation extraction have focused on sentence-level analysis, limiting their scope to single relations. While this is a valuable strategy, it fails to capture the complexity and depth of relationships present in the biomedical literature. The introduction of the BioRED dataset provided a more comprehensive and challenging framework (27, 44). BioRED extends beyond single, sentence-level extractions to encompass multiclass relation classification, including the identification of novel relationships.

Central to the process of automated relation extraction is the need for accurate Named Entity Recognition (NER) and entity normalization. Effective relation extraction cannot take place without these initial steps. NER involves identifying mentions of biomedical entities within the text, while entity normalization aligns these mentions with unique identifiers in a standardized vocabulary, ensuring that entities are consistently recognized across different documents (53). These steps establish a foundation from which meaningful relationships between entities can be identified and analyzed. These underscore how NER, entity normalization and relation extraction can be linked to the automated analysis of biomedical literature.

Considering these factors, we propose an innovative end-to-end system designed to address the present challenge of multiclass relation classification. This system is built upon a cascading pipeline framework, seamlessly integrating three specialized modules: (i) the “Tagger” dedicated to NER; (ii) the “Linker,” tasked with assigning entities to the standard vocabularies; and (iii) the “Extractor” focused on relation extraction and novelty detection. Delving deeper, our “Tagger” follows state-of-the-art methodologies by training a transformer-based model with the Masked Conditional Random Field (CRF) (29). For entity linking, we adopted a dual-searching approach by combining exact match with semantic search to find codes over the standard vocabularies. Lastly, for the “Extractor,”, we propose a joint model capable of simultaneously predicting relations while also assessing their novelty. This joint approach boasts significant efficiencies, notably eliminating the redundancy of multiple models.

Our system, named Biomedical Novelty Extractor (BioNExt), was initially tested during the BioCreative VIII Track 1 (BioRED) challenge (3, 27). The challenge was structured around two principal tasks: (i) relation extraction and novelty detection and (ii) end-to-end relation extraction and novelty detection. Initially, our efforts were focused on the first task, leading to the development of our “Extractor” module. However, this paper represents an extension of this preliminary work to also address the second task of this challenge. We have broadened the scope of our system to include the “Tagger” and “Linker” modules, thereby enhancing the capabilities of the system to identify all relationships and showcasing a comprehensive solution to the demands of advanced biomedical text mining. In summary, our main contributions in this paper are the following:

- An end-to-end model capable of identifying six types of entities, normalizing them to standard knowledge bases, extracting relations between the entities and classifying these relations as novel. Furthermore, we release the full model pipeline as an open source, enabling users to easily run it locally: https://github.com/ieeta-pt/BioNExt.
- To the best of our knowledge, we present the first exploratory usage of Large Language Models (LLMs) as few-shot learners for performing sequence variant annotation.
- The introduction of an innovative training methodology that simultaneously addresses the learning of relations and novelty.

## Background

Driven by the exponential growth of biomedical literature, the use of Natural Language Processing (NLP) for biomedical knowledge discovery has increasingly become a focal point of scientific research. The main goal of this domain focused on extracting insights from unstructured data, which can further the understanding of complex biological systems, disease mechanisms and potential therapeutic targets. The nature of biomedical knowledge increases the complexity of this task. However, it can be addressed in three critical components: NER, entity linking and relation extraction and classification.

### NER

In the biomedical field, NER aims to extract structured information from the extensive corpus of unstructured texts. This process involves the identification and categorization of key biomedical entities, such as genes, diseases and chemicals. Formally, given a sequence of tokens $s = w_1, w_2, {\ldots}, w_n$, the objective of NER is to generate a series of tuples $(I_s, I_e, t)$. Each tuple represents a named entity found in *s*, where $I_s, I_e \in [1,n]$ denote the starting and ending indices of the entity within the sequence and *t* corresponds to the type of entity, categorized according to a predetermined set of types (15).

Traditional, more straightforward strategies use dictionaries and regular expressions to identify entities in text (12). With the advent of machine learning, more sophisticated approaches were proposed, where NER was framed as a sequence labeling task, where each token is labeled as part of an entity (35). This fosters the early adoption of classification models into NER, also allowing for the detection of longer entities spanning multiple words.

The effectiveness of these sequence labeling models is further bolstered by their use of tagging schemas and sequence classification techniques. The beginning, inside, outside (BIO) schema is widely adopted due to its simplicity in marking the start, continuation and nonentity portions of text. Other tagging variants are known as the BILOU (beginning, inside, last, outside, unit) or IOBES (inside, outside, beginning, end, single) schema. These distinguish the last token of multitoken entities and single-token entities. Some authors report better NER results when employing a more detailed IOBES scheme (14, 58), whereas others did not observe significant improvements over the BIO tagging scheme (35). The BIO scheme remains as the most commonly used tagging scheme in the NER literature.

Neural networks have been heavily used in combination with CRFs for NER. Particularly, the use of bidirectional long short-term memory networks has been extensively used in the literature (11, 24, 35). With more details, CRF classifiers add another layer of sophistication to NER models (76). By considering the dependencies between sequential tags, CRFs ensure the logical coherence of identified entities commonly employing the BIO tagging scheme. In other words, these models take into account the previous predictions when making the next prediction in a (token-level) sequence.

The evolution of computational techniques has significantly advanced the state-of-the-art in biomedical NER, especially by the integration of transformer-based models, which enabled more accurate entity recognition (2, 32, 65).

Ensemble methods and postprocessing rules represent additional strategies to enhance NER accuracy (8). By aggregating predictions from multiple models or iterations, ensemble methods can mitigate individual model biases or errors, leading to more reliable entity recognition. Postprocessing steps, which apply domain-specific heuristics, further refine the model’s output, correcting common mistakes and resolving ambiguities inherent in biomedical entity recognition.

The NER-detected entities are generally domain-specific or can be common entities. For example, in the biomedical domain, an entity can refer to diseases or chemicals, or in the common domain, this can refer to people, places or objects.

In 2023, National Center for Biotechnology Information (NCBI) released AIONER, a state-of-the-art tool for recognizing entities of different types at once, resulting in improved robustness (45). They proposed the all-in-one tagging scheme to accept different entity classes from multiple datasets. This tagging schema was trained upon several datasets, including the BioRED dataset.

### Entity linking

Named entity linking or named entity normalization refers to the task of assigning unique identifiers to named entities from standard terminologies. This step is usually followed by the NER task where entity text mentions are already detected within a text. In the biomedical domain, there are several knowledge resources to aid entity linking for different entity types such as genes and diseases with many of these terminologies being included in the Unified Medical Language System  (9). For example, NCBI Gene contains information on genes (10), and the Medical Subject Headings (MeSH) vocabulary contains unique identifiers for biomedical and health-related concepts including diseases and chemical substances (42). These vocabularies are commonly employed to link entities to their unique identifiers, which is helpful for downstream tasks such as relation extraction.

Different shared tasks have been conducted, and several datasets have been published throughout the years for biomedical entity linking due to its inherent importance and difficulty (21). Regarding the linking of entities found in the biomedical scientific literature, BioCreative has been the foremost effort having organized multiple challenges since 2004 spanning entity normalization for various biomedical concepts (26, 36, 75). In the clinical domain, 2019 n2c2 Track 3 showcased a challenge to normalize medical concepts (problems, treatments and tests) using Systematized Nomenclature of Medicine Clinical Terms (SNOMED CT) and RxNorm vocabularies (47). Similarly, ShARe/CLEF 2013, SemEval-2014 Task 7 and SemEval-2015 Task 14 shared tasks focused on normalization of disorders being mapped to SNOMED CT (20, 55, 56). Identical challenges have been organized by the Text Mining Unit at the Barcelona Supercomputing Center where entities of different types, found in Spanish clinical narratives, are normalized to SNOMED CT: PharmaCoNER for pharmacological substances (22), DisTEMIST for diseases (51), MedProcNER for medical procedures (40) and SympTEMIST for symptoms (41). The SMM4H 2017 shared task tackled normalization of adverse drug reactions, found in social media text, to MedDRA concepts (61).

Traditional approaches for entity linking rely on dictionaries or gazetteers, containing a list of terms mapped to their unique identification, where direct string matching, regular expressions or heuristics are employed. More advanced methods create numerical representations (embeddings) for the entity mentions and the terms found in standard vocabularies associated with unique identification codes. The aim is then to compute a concept vector for a given entity mention and identify the nearest vector among all concepts in the vocabulary. Typically, the comparison of these vectors is achieved through cosine similarity. Construction of these concept vectors typically relies on the shallow neural model or transformer-based language models such as SapBERT for text representation (43, 50). State-of-the-art tools for NER and normalization of multiple biomedical entities include BERN2 and HunFlair2 which rely on transformer-based models (60, 66).

With special focus on the biomedical domain, NCBI in 2012 published PubTator as a tool for helping with manual curation such as annotating biomedical concepts in PubMed abstracts (71, 72). Since 2013, PubTator provides preannotations on different biomedical concept types with state-of-the-art performance for assisting manual biocuration (73). Over previous years, PubTator has been an evolving web-based text mining system for assisting biocuration and has been shown to improve both efficiency and accuracy of manual curation. In 2019, improved upon its predecessor, PubTator Central was published and allowed us to retrieve and view bioconcept annotations in PubMed abstracts and full-text articles with a renewed web interface (69). It used state-of-the-art text mining systems for identifying six concept types: genes (proteins), genetic variants (mutations), diseases, chemicals, species and cell lines.

Recently, PubTator 3.0 was released with numerous improvements including not only entity annotations but also semantic relationships (68). Its entity recognition performance was also improved when comparing to PubTator Central (also known as PubTator2). PubTator 3.0 includes 12 relations types such as association, cause, drug interaction, inhibition, stimulation, treatment and others. Their entities are identified using the previously proposed AIONER system (45) and are linked to standard vocabularies using a variety of tools: GNorm2 is used to normalize genes to NCBI Gene and species to NCBI Taxonomy (74), TaggerOne normalizes diseases to MeSH and cell lines to Cellosaurus (37), chemicals are normalized using the NLM-Chem tagger to MeSH identifiers (28) and tmVar3 normalizes genetic variants using NCBI dbSNP identifiers (rs#) or tmVar normalized forms (70).

### Relation extraction and classification

Biomedical relation extraction involves identifying semantic relationships between entities mentioned in biomedical texts. The primary goal of relation extraction is to uncover pairs of entities within the text that exhibit some form of semantic connection. These entities could represent various biomedical concepts such as proteins, genes, diseases, chemicals and their various interactions. It is worth noting that, although typically a relation is marked between two entities, a relation can involve more than two entities. Overall, relation extraction plays a pivotal role in tasks such as knowledge base construction, information retrieval and biomedical literature mining (25, 30).

Relation classification builds upon the extracted pairs of entities by aiming to discern the specific type of relationship that exists between them. Once the entities participating in a relation have been identified, relation classification seeks to categorize these relations. In the context of biomedical texts, these relations can encompass a wide range of interactions, such as protein–protein interactions (33), drug–disease associations (75), chemical–protein interactions (52) and more. Effective relation classification algorithms leverage machine learning techniques, often utilizing annotated datasets to train models capable of accurately predicting the relationship types between entities. Classical rule-based approaches (7) in biomedical relation extraction have gradually been surpassed by more sophisticated deep learning methodologies. The advent of transformers and models like Bidirectional Encoder Representations from Transformers (BERT) (18) has led to a paradigm shift, with the majority of challenges in NLP, including relation extraction, now being predominantly addressed using transformer-based architectures. Notably, transformer-based models have demonstrated state-of-the-art performance in various relation extraction tasks (78). Additionally, there has been notable research on jointly performing NER and relation extraction (1, 6, 19).

While traditional approaches often treated relation extraction and classification as distinct tasks, the advent of deep learning has facilitated the integration of these tasks into a unified relation classification framework. This integration is achieved by introducing a negative class into the relation classifier and eliminating the need for a separate relation extractor (3). By doing so, the model is trained to not only detect relations but also classify them.

Research on novel relation classification in biomedical text mining is relevant for biomedical researchers to stay updated about new discoveries (27, 44). Some approaches aim to perform relation classification and novelty detection simultaneously, integrating these tasks into a single step (38, 48). In contrast, others focus on classifying predefined relations while explicitly seeking to identify and classify novel relationships between entities (13, 34, 39, 49, 54, 59, 62).

## Methodology

In this section, we describe the dataset, the evaluation metrics used for this task and all the details regarding the end-to-end relation extraction and novelty detection model.

### Dataset

The BioRED dataset (44) spans six distinct entity classes: Genes, Diseases, Chemicals, Variants (mutations), Species and Cell Lines, with the goal of revealing previously undiscovered interactions between these entities. The dataset was curated from PubMed documents selected via specific queries. A dedicated team of three annotators with a biomedical informatics background undertook the initial annotation of entities and relations. The annotation of these documents was conducted using PubTator3, with annotation being conducted before publication in 2022. Moreover, the task of discerning novelty among these relationships was entrusted to two biologists, ensuring the validity and significance of the associations identified. Initially, the dataset consisted of 600 documents, which were divided into sets for training (400), validation (100) and testing (100). For the competition phase (BioCreative VIII Track 1), an additional 400 documents were introduced as a blind test set (27), with the expanded dataset’s annotation responsibilities being carried out by eight biocurators from the National Library of Medicine. Within the BioRED dataset, each of the six entity classes is linked to its respective standard vocabulary as specified below:

- Gene: NCBI Gene (10)
- Disease: CTD diseases (16, 17)
- Chemical: MeSH (42)
- Variation: dbSNP (64) and tmVar (70)
- Species: NCBI Taxonomy (63)
- Cell lines: Cellosaurus (5).

Additionally, the authors identified a total of eight possible relationships between the entities, namely, Positive Correlation, Negative Correlation, Association, Binding, Drug Interaction, Cotreatment, Comparison and Conversion. The relationships predominantly feature interactions among Diseases, Genes, Variants and Chemicals, mirroring their frequent occurrence in the biomedical literature. Table 1 provides a detailed breakdown of the distribution of entity mentions throughout the BioRED-BioCreative VIII (BCVIII) dataset, as well as the interactions between entities.

**Table 1.** BioRED-BCVIII annotation statistics and, in parentheses, the unique set.

Annotations	Train	Test
Documents	600	400
Gene	6697 (1643)	5728 (1278)
Disease	5545 (778)	3641 (644)
Chemical	4429 (651)	2592 (618)
Variant	1381 (678)	1774 (974)
Species	2192 (47)	1525 (33)
Cell Line	175 (72)	140 (50)
Total	20,419 (3869)	15,400 (3597)
Disease–Gene	1633	1610
Chemical–Gene	923	1121
Disease–Variant	893	975
Gene–Gene	1227	936
Chemical–Disease	1237	779
Chemical–Chemical	488	412
Chemical–Variant	76	199
Variant–Variant	25	2
Total	6502	6034
Novel Relations	4532	3683

### Evaluation metrics

The official evaluation metrics used in this work are micro-average Precision, Recall and F1-score (main evaluation metric). These metrics take into account the number of True Positives (correct predictions), False Negatives (incorrect negative predictions) and False Positives (incorrect positive predictions). The BioCreative VIII BioRED challenge was organized in two subtasks. In Subtask 1, participants were given PubMed abstracts, with annotated entities by human experts, and were asked (i) to extract relation pairs, (ii) to identify their semantic type and (iii) whether the relation is novel. The annotated entities included the text mention (span with start- and end-character offsets), the entity type (gene, disease or other) and an identifier code linked to a specific terminology (NCBI Gene, MeSH or others). In Subtask 2, participants were solely given the PubMed abstracts and were challenged to build an end-to-end system for the same relation extraction task (identification of relation pairs, relation classification and their novelty factor).

The organizers considered four evaluation results for relation extraction:

- **Relation pair identification**: Whether a pair of entities was identified as constituting a relationship;
- **Relation classification**: Whether an entity pair relationship was categorized with the correct relation class (association, drug interaction, positive correlation or others);
- **Relation novelty factor**: Whether an entity pair relationship is considered as novel given the article context.
- **All**: This includes all the previous three scenarios, a relation is considered to be correctly predicted if an entity pair relationship exists and it is classified with the correct relation type and the correct novelty factor.

These four scenarios were evaluated, and teams were sorted according to each of these results for relation extraction. In Subtask 2, participants needed to build their NER and entity linking systems and these tasks were also taken for evaluation with the microaverage F1-score. During the development of our end-to-end model presented in this work, we also calculated the NER and entity linking results per each entity class to inspect which entity classes needed more refinement and attention from our model.

### End-to-end system

**Figure 1.**
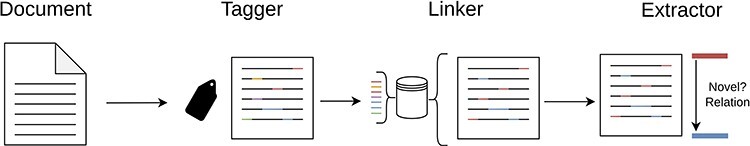
An overview of our cascade pipeline, which showcases the interaction between the three main modules: Tagger, Linker and Extractor.

As previously mentioned, our system is structured around three core modules—“Tagger,” “Linker” and “Extractor”—which operate in a cascading pipeline, a brief architectural overview can be seen in Figure 1. Each module is designed to address, in an isolated manner, a distinct task within the broader process.

- **Tagger (NER)**: The “Tagger” module’s objective is to identify biomedical entities within a document, classifying them into one of several categories: Gene, Disease, Chemical, Sequence Variant, Species (Organism) or Cell Line.
- **Linker (Entity Linking)**: Following entity identification, the “Linker” module takes over to normalize the identified entities to their corresponding entries in standard knowledge bases, thus ensuring consistency and accuracy in entity representation.
- **Extractor (Relation Detection and Classification)**: The final module, “Extractor,” is tasked with discerning the relationships that exist among the various entities within a document. It classifies these relations into one of the eight predefined categories and identifies which of these relations are novel, i.e. not previously described in the literature.

Our system is developed entirely using Python, with the use of PyTorch and Hugging Face libraries for the implementation of the various models models (more information is provided in the GitHub repository^1^). All the code run on a machine with Intel(R) Xeon(R) Gold 5218R CPU, 128GB of RAM and a NVIDIA Quadro RTX 8000.

#### Tagger (NER)

**Figure 2.**
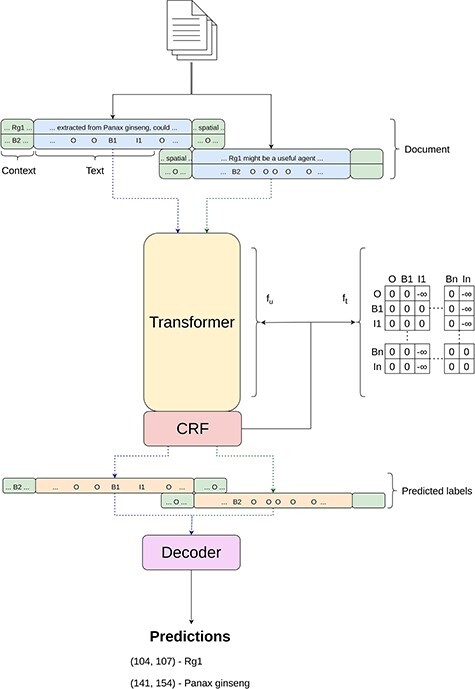
Simplified overview of the inner workings of the Tagger module.

For the “Tagger” module, our approach aligns with methodologies described in other works (3, 4, 44), which can be seen in Figure 2, employing the BIO-tagging schema for data encoding. This schema, widely adopted in the field as highlighted by Lample *et al.* (35), assigns each token to a category—beginning (B), inside (I) or outside (O)—to mark entity boundaries within the text. Subsequently, data encoded in this manner are processed by a transformer-based model, which is enhanced with a Masked-CRF classifier (2, 76), to accurately identify entity types.

Given the variety of entities in the BioRED dataset—ranging from Genes and Diseases to Chemicals and Cell Lines—the need for a multiclass approach becomes evident, which differs from previous works that primarily focused on single-entity identification. To accommodate this, we extend the BIO tagging schema to multiple classes. As a result, our label set expands to include specific tags for each entity class, such as B-Gene, I-Gene, through to B-Diseases and I-Diseases, framing this as a 13-class sequence classification problem.

Formally, let us consider $x=\{x_1,x_2,{\ldots},x_N\}$ as a sequence of text tokens, where *x*_*i*_ represents the *i*-th token in the text and *N* denotes the total number of tokens; $y=\{y_1,y_2,{\ldots},y_N\}$ as the corresponding sequence of labels, where each *y*_*i*_ from a set of predefined labels such as $\{O, {\rm B-Gene}, {\rm I-Gene}, {\ldots}, {\rm B-Diseases}, {\rm I-Diseases}\}$ is assigned to token *x*_*i*_. To estimate $P(y|x)$, the probability of assigning a label sequence *y* to a given token sequence *x*, traditional methods might assume label independence, calculating $P(y|x)$ as a product of individual label probabilities given the entire sequence, $P(\textbf{y}|\textbf{x}) = \prod^N_{i=1} P(y_i|\textbf{x})$. However, as pointed out in (44), this overlooks the dependencies between labels, especially critical in BIO tagging where, for example, an “I” (inside) tag must always follow an “B” (beginning) tag. To account for label dependencies, we modify the approach to include the probability of each label not just given the entire sequence *x* but also considering the previous label, thus incorporating sequential context. In practice, this context-aware estimation is achievable by using linear-chain CRF to model $P(y|x)$,


$$ P(y|x) = \frac{1}{Z(x)}\exp{\left (\sum^N_{i=1} f_u(y_i,x;\theta_u) + \sum^{N}_{i=2} f_t(y_i,y_{i-1};\theta_t) \right )},\\[5pt] $$

where *θ* represents the trainable parameters, *f*_*u*_ is the unary function and *f*_*t*_ represent a transition function. The unary function computes the unary potentials which essentially compute the score of each label being assigned to token *x*_*i*_ while considering the whole sequence. *f*_*t*_ is the transition function that simply corresponds to a transition matrix, being parameterized by *θ*_*t*_ and having its score obtained by looking up the entry in the matrix. Lastly, $Z(\textbf{x})$ is known as the partition function and acts as a normalizing factor to obtain a probabilistic distribution over all sequences.

Additionally, to further enforce the restrictions of the BIO tagging schema, we follow the ideas of (3, 44, 76) and masked our CRF by applying a large negative weight to the impossible transitions according to the BIO schema, like predicting a “I” after an “O.”

We relied on BERT-based (18) encoders as part of the unary function of the Masked CRF. Given the limited context size of this architecture (512 tokens), we also adopted a sliding window strategy to split the document into more manageable sizes. More precisely, we consider a window of size of 512 tokens, but with *k* tokens as left and right context. These context tokens are not taken into consideration for the final label prediction, but are rather for contextualizing the actual predictions.

During training, we also adopted the “Random Token Replacement with Unknown” augmentation technique presented in (3). This involves randomly replacing entities tokens in the input sequence with the special unknown token “[UNK].” The intuition is to force the model to not only rely on the entity text but also use the context tokens.

Finally, we employ two “postprocessing” steps, namely, decoding and ensembles. The decoding phase takes the label outputs from the various sequences for each document and extracts the corresponding entity class and spans. The ensemble consists in combining the outputs of various models at the entity level, taking advantage of the knowledge learnt by multiple models.

#### Linker

To perform entity linking on the entities identified from NER, we employ a multistage pipeline in an attempt to maximize the number of hits for the entities detected in the previous stage of the model. Although we attempt to incorporate the same linking methodology, in practice, most of the knowledge bases have significant differences between them and hence require a different pipeline to process them. Furthermore, for each entry in a knowledge base, there may be many ways to find a code (by looking for concepts, synonyms, or description), and so a single text term can correspond to many identifiers, which creates a problem of disambiguation.

**Figure 3.**
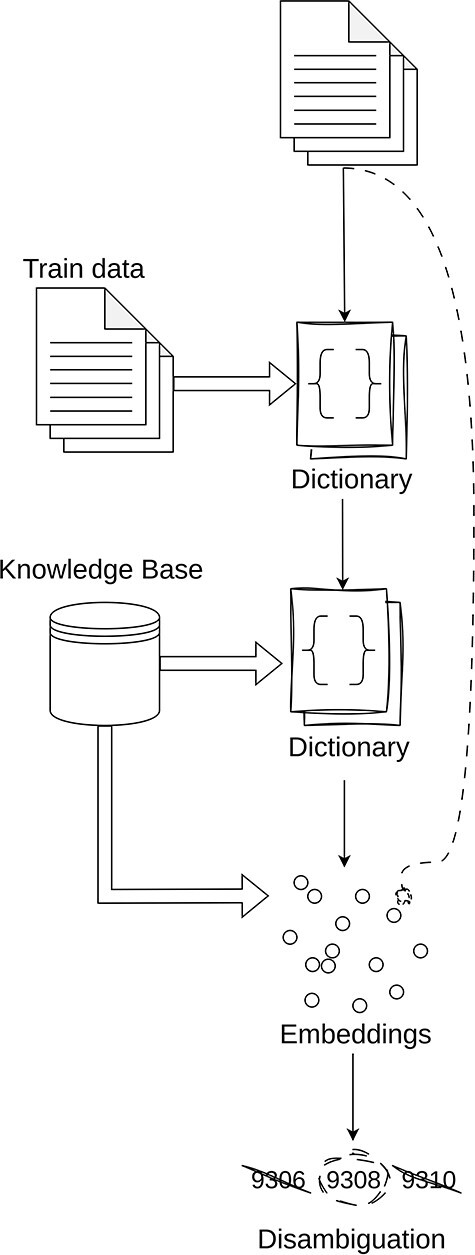
Simplified overview of the inner workings of the Linker module.

The general idea behind our entity linking is as follows (illustrated in Figure 3):

- **Direct match over training data:** The initial step in our pipeline is to create a dictionary of the training entities and their corresponding codes. It is assumed that any entity that exists in the training data can be assigned its corresponding code.
- **Direct match over corpus:** The next step is to perform direct matching over the respective knowledge corpus. The step has unforeseen challenges such as the large scales of the various corpora as well as the number of sources for which direct lookup can be performed. Details regarding this will be provided in the detailed explanation of each knowledge base.
- **Semantic search over the corpus:** The next step is to perform a semantic search using text embeddings from SapBERT (large) (43). When using the embeddings, we perform cosine similarity between the corpus and the entity text. In the cases where we have multiple text knowledge bases, we select the code that has the largest cosine similarity value above a certain threshold.
- **Disambiguation:** The final step is to resolve ambiguities in terms that have multiple codes assigned to them. For this, we propose a naive algorithm that selects the most frequent code that is shared between the maximum number of entities. This approach is based on the premise that documents are likely to maintain consistency in their annotations, suggesting that the most shared code among entities is the most likely to be the correct one.

When performing linking, unless otherwise stated, it is assumed that all the entity terms are converted to lowercase in order to maximize matching between entity mentions and our vocabulary entries.

##### Species/organism

To normalize species, we use the NCBI-Taxonomy knowledge base. In this corpus, there exist 2,564,321 codes, containing a total of 3,998,949 terms which we use to build the dictionary for test. These values are from the “name_txt” field from the names.dmp file, which can be obtained here.^2^ For the linking of species, we only relied on direct match on training, direct match over the corpus and disambiguation. We did not employ any semantic search here, due to the higher matching scores that we were obtaining with direct match (97%).

##### Chemicals

For the linking of chemicals, we utilized MeSH codes specifically associated with chemicals, incorporating both the official codes and those from the Supplementary Concept Records. To elaborate, we filtered the codes starting with “D*,” which are explicitly designated for chemical substances within the MeSH hierarchy. For our search, we considered only three fields, namely, concepts (25,465) synonyms (93,091) and definitions (10,812). These correspond to 10,541 different entities. The supplementary data contain an additional 323,495 entities and 216,429 definitions presented. After performing the exact match on the training, we then perform the semantic search over the embeddings of both concepts, synonyms and definitions combined, selecting the highest cosine score over a threshold.

##### Diseases

Diseases follow a similar methodology as chemicals in terms of the linking pipeline. However, as knowledge bases we used the CTD disease corpus, containing MeSH and the Online Mendelian Inheritance in Man database. Again, we use the three fields, namely, concepts (13,298) synonyms (77,319) and definitions (10,812). There exist 13,298 unique codes in the MeSH corpus for diseases, which were filtered with descriptors starting with C*.

##### Cell lines

In the case of the Cell lines linking, we utilized the Cellosaurus knowledge base, which contains 152,231 unique codes, each associated with a distinct concept. In terms of linking, we follow the standard pipeline, by performing direct match over training and corpus and then using semantic search to find semantic similar matches on the remanding of terms.

##### Genes

For gene linking, we relied on the NCBI-Gene knowledge base that assigns unique codes to organisms associated with specific genes. A crucial aspect of this process is recognizing that a gene’s identity is contingent upon its organism, given that identical genes may exist across different species. To address this, our preliminary step involves identifying the relevant organism for each gene mention in the document. For this, we implemented a straightforward algorithm that looks up up for the closest organism mention to the gene term directly in the text. Here, our assumption is that the closest organism to a gene mention must be the organism that the gene is referring to. Additionally, if no organism is found, we consider the organism to be human (code 9606).

Following this organism identification, we continued with our previously described linking methodology, but now by only considering gene codes that belong to the identified organism. With respect to the NCBI-Gene knowledge base, there are 48,880 organisms with genes, totaling 50,941,500 unique gene codes. For each, we only consider the fields, symbols, synonyms, descriptions and other designations as descriptors totaling 101,928,344.

Finally, due to computational requirements, our semantic search with embeddings is limited to the top seven organisms most prevalent in the corpus: house mouse (10 090), Norway Rat (10 116), human immunodeficiency virus (11 676), Respiratory syncytial virus (12 814), thale cress (3702), zebrafish (7955) and human (9606). This selection was necessary due to the substantial memory requirements for holding the embeddings, which would exceed the 300 GB for the full knowledge base.

##### Sequence Variants

Sequence variants present a unique challenge within our pipeline, primarily because most do not have a unique identifier and instead utilize the tmVar (70) code notation. This notation is a manually crafted format that standardizes the description of sequence variants. For example, “Arg-114” and “-3170G>A” are standardized to “p$\vert$Allele$\vert$R$\vert$114” and “c$\vert$SUB$\vert$G$\vert$-3170$\vert$A,” respectively. Additionally, similar to gene linking, the sequence variants can have different codes depending on the gene where they are expressed. Given these circumstances, we divided the linking task in three stages. First, we identify the gene each sequence variant refers to; for that, we use the same algorithm used in gene linking to find the nearest gene mention to the sequence variant. Next, we conduct a direct lookup for the sequence variant and gene within the dbSNP database. If this search is unsuccessful, we then proceed to generate the corresponding tmVar code notation.

Regarding the direct lookup, our first approach was to download the entire dbSNP database, which catalogs genetic variation codes across various species. However, given the database’s substantial size (over 200 GB of raw data), we opted instead to utilize LitVar2, a public Application Programming Interface (API) capable of performing sequence variant lookups.^3^ Note that this API represents the only third-party dependency in our pipeline although theoretically, we could develop an in-house solution for sequence variant lookup.

With regard to the tmVar notation generation, we initially tried to use tmVar itself; however, we were unable to make a successful deployment of the tool. Therefore, we propose to frame this task as a translation problem, where we trained a translation model and also tried a few-shot LLM generation approach.

Regarding the generation of tmVar codes, we aimed to approach this task as a translation problem. The underlying idea is to translate a given gene and sequence variant mention into the corresponding tmVar notation. Specifically, we investigated training a translation model and utilizing a LLM for few-shot code translation. For the translation model, we utilized the plain T5-base model trained on pairs of sequence variants and tmVar notations from the training dataset. Meanwhile, for the LLM approach, we conducted a semantic search to identify up to 25 similar translation examples from the training data. These examples were then used to instruct the LLM to generate the next code, following the patterns observed. The detailed prompt used is presented in Prompt 1. Additionally, we observed that amino acids and their respective codons are typically normalized to their corresponding single-letter codes. To accommodate this, we manually constructed a translation table that converts both codons and amino acids into their single-letter representations.

**Prompt 1.**
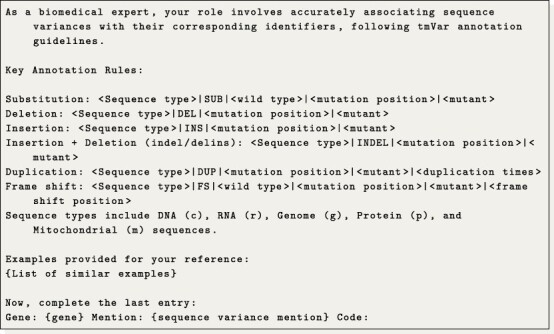
Example of the prompt used to translate codes under a few-shot configuration.

#### Extractor

As mentioned, the objective of the “Extractor” is to identify relations between the normalized entities, classify them and determine which of these relations are novel. Most of our “Extractor” module follows the system already presented in the Biocreative VIII BioRED track (3), with a small set of additions and corrections. To make this work self-contained, we will briefly describe our previous “Extractor” model, and then we will discuss the changes that we made.

First, let us define the task of relation extraction as assigning potential relations $r_k\in R$ to pairs of entities $(e_i,e_j)$ within a document *D* containing *E* unique linked entities, culminating in the triplet $(e_i,r_k,e_j)$. Additionally, we can also frame the novelty task as a binary classification task over the previous triplet $(e_i,r_k,e_j)\rightarrow \{0,1\}$. However, while a document may theoretically contain up to *E*^2^ entity pairs, in reality, only a smaller subset of these pairs have relevant relations. To address this, we introduced an additional “negative” class alongside the original set of eight classes, specifically to identify instances where the entity pair does not exhibit a relation. Based on this joint definition, we can now develop a model capable of simultaneously predicting relations while also assessing their novelty for any given document annotated with a set of $(e_i,e_j)$.

**Figure 4.**
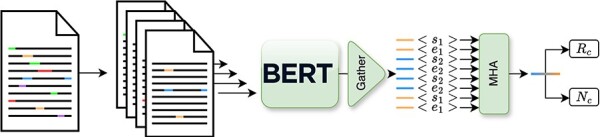
Simplified overview of the inner workings of the Extractor module, as depicted in previous work (3).

In terms of architecture, as depicted in Figure 4, the model leverages a transformer-based architecture to produce contextualized representation for each entity. Then from these, we produce, in a multihead attention layer, a joint representation that we use to perform both the relation classification and novelty detection. Furthermore, to accurately encode contextual entity information as input for the model, we introduce new tokens “[s1],” “[e1],” “[s2],” and “[e2],” which correspond to the start and end of the two entities in the text. These tokens are then directly inserted into the text. For example, in the sentence “(…) high-grade [s1]glioma[e1] (…),” “glioma” corresponds to the first entity. In order to jointly train this model on both tasks, we propose a masked combined loss defined in Equation 1,


(1) $$ L=L_{\rm r} + (y_{\rm r} \neq 8)L_{\rm n}, $$

here, we sum the cross-entropy loss for the relation (*L*_r_) and novelty (*L*_n_) losses. Notably, the novelty loss *L*_n_ is considered only when the entity pair is deemed valid, i.e. its relation, *y*_r_ does not correspond to the negative class $(y_{\rm r} \neq 8)$.

Building on our initial model, we have introduced some postchallenge enhancements that will be discussed later.

- **Dynamic negative sampling:** In our previous approach, we randomly selected negative examples from the training data, where a negative example corresponds to an entity pair without a valid relation. Due to the vast number of possible pair combinations, many of these negative pairs represented easy cases that offered minimal contribution to model training. To address this issue, we introduced a strategy for dynamically sampling negative examples using a previously trained model. With more details, initially, we train a model using a random negative sample, which we call of *M*_0_. Next, we generate a new dataset of negative samples by applying *M*_0_ to the training data, focusing on selecting pairs for which *M*_0_ showed low confidence in negative classification or incorrectly predicted as positive. The rationale here is that these examples should correspond to “harder” negative pairs that when prioritised over easy negative samples should yield better training performance. This curated set of “harder” negative examples is then used to train a new model, referred to as *M*_1_. It is worth noting that this process can be iteratively repeated to generate further model iterations although computational costs increase significantly with each cycle. Exploring the benefits of continued iterations was left for future work.
- **Correction of an assumption:** In our previous work, we assumed that the relation triples were directional, in that $(e_i,r_k,e_j) \nRightarrow (e_j,r_k,e_i)$. However, we later discovered that this statement is incorrect and that $(e_i,r_k,e_j) \Rightarrow (e_j,r_k,e_i)$, which also reduced the number of negative samples present in the dataset.

## Results and discussion

In this section, we focus on evaluating and discussing the outcomes achieved by our individual modules and the integrated end-to-end system. Initially, we present the results obtained on the validation set. Subsequently, we detail the performance of our model on the test set and offer a comparative analysis with our submissions to BioCreative VIII Track 1 Subtask 2.

It is important to note that there is not an official evaluation script provided and the primary mode of evaluation is through a CodaLab^4^ competition set up by the event organizers. However, this CodaLab competition does not offer metrics for NER or Linking as it primarily focuses on Subtask 1. Consequently, the results we report for NER and Linking were derived using our own evaluation scripts. Due to this discrepancy, we refrain from comparing our NER and Linking outcomes directly with those from BioCreative as we cannot guarantee the consistency of our metrics with those used by the organizers. Instead, we benchmark our NER and Linking performances against PubTator 3 (68), which represents the current state-of-the-art for both tasks.

### Validation results

Here, we first discuss the performance of each module individually and then conclude with the cumulative results of all modules combined in an end-to-end fashion. All the measures are reported over the validation set, containing a total of 100 documents.

#### Tagger

For the “Tagger,” we are mainly concerned to evaluate the NER performance of our models. In terms of configuration, we adopted the BioLinkBERT-large model, mainly due to the superior performance showcased during the challenge versus other pretrained models. Furthermore, we adopted a context size of 32, while not noticing any difference to other context sizes. We believe that this is mainly explained by the documents being only abstract, and in most cases, they fit inside the initial window size, hence not being necessary to split. Regarding training, we mainly stick with the default hyperparameters present on the Hugging Face (77) trainer, with the addition of using the unknown random augmentation technique previously described.

Regarding the results, we present in Table 2 the performance of our NER model versus the state-of-the-art PubTator 3, in terms of micro-F1 score. With more details, we trained, with different random seeds, and evaluate five NER models and report the results as the average and as a single run produced by an entity-level ensemble over our five runs. Compared to PubTator 3, our model demonstrates significantly superior performance, outscoring it by 12.46 points. This is an interesting result considering that PubTator 3 was trained on a larger and more diverse datasets, including the BioRED dataset, showing the importance of fine-tuning on domain-specific data, as varying annotation guidelines across datasets can lead to inconsistencies in entity recognition, which we believe being the main reason for these differences. Additionally, it is compelling to note that our entity-level ensemble method managed to produce a combined run that exceeds the average scores of all individual runs. This suggests that leveraging multiple models in an ensemble can effectively enhance overall performance.

**Table 2.** Comparison of five runs best performing model with PubTator 3.

	BioNExt	BioNExt	
Entity	(average)	(entity ensemble)	PubTator 3
Gene	92.54 ± 0.56	**93.16**	68.42
Disease	84.80 ± 0.46	**85.97**	79.74
Chemical	91.87 ± 0.41	**92.33**	86.04
Variant	85.49 ± 0.73	**85.88**	82.28
Species	89.91 ± 0.54	**90.21**	80.61
Cell Line	91.40 ± 1.80	**91.84**	80.77
Total	89.57 ± 0.21	**90.24**	77.68

Results are presented in terms of F1 score. The best scores are highlighted in bold.

#### Linker

Before addressing the main results, let us discuss our methodology for generating tmVar codes. As previously mentioned, we propose two strategies: (i) training translation models and (ii) utilizing a LLM with a few-shot prompt. For the translation models, we trained both T5-small and T5-large models (57) on tmVar codes from the training data and the tmVar 3.0 corpus (70). For the LLM strategy, we employed the hermes-2-mixtral (67) model. In terms of performance on the validation set, the best translation model achieved an accuracy of 44.15%, while the few-shot LLM approach reached 69.37%. Based on these outcomes, we opted for the LLM approach when predicting tmVar codes.

Regarding the main results, it is important to note that the results for linking are obtained over the previous NER runs. The main reason for this is because we were not capable of run PubTator 3 over the gold-standard entities due to the unavailability of the API. So it should be mentioned that given our superior NER results, we expected to have an advantage in this linking stage. We further would have liked to test out our NER model with PubTator’s linking; however, the API service was not functioning at the time.

However, as observable by the results presented in Table 3, our entity linking performance falls short of PubTator 3, trailing by almost 5 points (77.05 versus 81.96). A closer examination of the results reveals that the discrepancy is most pronounced in the Gene class, with a nearly 10-point gap. This poor performance on the gene class was somewhat anticipated, given that it depends on finding the correct species that it belongs to. Consequently, any error in the linking of species will directly impact the linking of genes, which aligns with our comparably poor species performance. In light of these unexpected results, a considerable part of the Error Analysis section is dedicated to a more thorough examination of these findings.

**Table 3.** Entity linking comparison of our best performing model with PubTator 3.

Entity	BioNExt	PubTator 3
Gene	74.85	**84.84**
CellLine	72.22	**80.85**
Variant	57.34	**60.08**
Chemical	83.64	**84.69**
Disease	78.86	**80.28**
Species	93.27	**97.76**
Total	77.05	**81.96**

Results are presented in terms of F1 score. The best scores are highlighted in bold.

Nevertheless, it is also important to consider that the PubTator 3 system applies different specialized state-of-the-art tools for normalizing each of the entities (68), namely, GNorm2 (74) for genes and species, TaggerOne (37) for diseases and cell line, NLM-Chem tagger (28) for chemicals and tmVar 3.0 (70) for variants. On the contrary, we focused on having a generic methodology that could be applied to any entity type, which eases the maintenance, since it is a single implementation.

Lastly, given the gene linking poor performance, we anticipated a significant impact on the “Extractor” module’s performance, as genes are involved in half of the relations according to Table 1.

#### Extractor

The evaluation of the “Extractor” module was conducted in the context of the Biocreative VIII Track 1 challenge. Here, we will discuss some validation results obtained during the challenge, as well present new validation results.

Primarily, regarding the type of transformer model to adopt, we mainly consider the two state-of-the-art BERT-based models BiomedBERT^5^ (23) and BioLinkBERT (79), as well as the new decoder-only BioGPT model (46). Note that our proposed model operates at the contextualized representation level, enabling compatibility with any type of transformer model. Table 4 presents the final entity pair and novelty score that we obtained for the validation set, when using different pretrained transformer models. As observable, our best results were obtained by using the BioLinkBERT (large) model, which aligns with the literature (79).

**Table 4.** The impact of pretrained transformer-based models as the backbone for the task of entity pairing and novelty discovering.

Pretrained model	Entity pair	+ Novel
BioLinkBERT (large) (79)	**75.99**	**53.43**
BioGPT (46)	61.64	40.59
BiomedBERT (23)	72.38	49.34

Results are presented in terms of F1 score. The best scores are highlighted in bold.

Regarding the postchallenge enhancement, we mainly propose the dynamic sampling strategy, which we now evaluate. Table 5 showcases the comparison when using dynamic sampling versus random sampling. As observable, by leveraging the dynamic sample, we were able to gain more than 2 points in terms of entity pair score, which then further translates to gains in novelty. This result aligns with our intuition, since the main reason for the dynamic sampling was to force the model to train on “harder” negative entities pairs. Furthermore, we only conducted experiments with a single iteration (*M*_1_) of dynamic sampling.

**Table 5.** Comparison between random sampling and dynamic sampling on entity pairing and novelty discovering.

Sampling strategy	Entity pair	+ Novel
Random sampling	75.99	53.43
Dynamic sampling (*M*_1_)	**77.76**	**55.37**

Results are presented in terms of F1 score. The best scores are highlighted in bold.

#### End-to-end

Lastly, we present in Table 6 the validation results for our complete pipeline. In this comparison, we assess the performance of our end-to-end system against the combined output of PubTator 3 and our “Extractor” model.

**Table 6.** Performance comparison of PubTator 3 + our Extractor with our end-to-end system, on validation data.

Configuration	PubTator 3	BioNExt
Configuration	+ Extractor (BioNExt)	BioNExt
Entity pair	**52.49**	43.95
Entity pair + Relation	**44.80**	37.60
Entity pair + Novelty	**43.10**	36.59
All	**36.69**	31.25

Results are presented in terms of F1 score. The best scores are highlighted in bold.

As anticipated, our comprehensive pipeline does not perform as well as PubTator 3. This outcome is primarily attributed to the subpar performance of our “Linker” in comparison to that of PubTator 3. Additionally, we believe that further exploring the integration of the PubTator linker with our NER could be beneficial. At the time of writing, we have attempted to use the PubTator linker exclusively with our NER outputs but have not succeeded.

### Submission results

In this section, we detail the performance of our systems on the final test set. As mentioned, given that the test set gold standard is not available, all evaluations were conducted using the CodaLab platform provided by the event organizers, limiting our metrics to the relation extraction task. Regarding the results, we begin by outlining our performance during the challenge, followed by a comparison with the postchallenge enhancements. Subsequently, we evaluate the performance of our end-to-end system in the relation extraction task, benchmarking it against PubTator 3.

#### Extractor

Table 7 shows the performance of our “Extractor” model when evaluated during the challenge. We submitted five runs, with the first two being both single models that utilized BioLinkBERT. The primary distinction between them was the seed used for negative random sampling. The remaining runs were ensembles of our top 8, 5 and 3 runs, respectively. Notably, Run 1 emerged as our best-performing run, closely followed by Run 4. The significant performance disparity between Runs 0 and 1 shows the influence of our negative random sampling approach, suggesting that Run 0 suffered from a less advantageous pool of negative documents, which likely contributed to its suboptimal results. This observation reinforces our rationale for adopting a dynamic negative sampling method, aiming to mitigate such impacts.

**Table 7.** Results of our five runs submitted to the challenge, as well as the median and average.

Configuration	Entity Pair (P/R/F%)			+ Relation (P/R/F%)			+ Novel (P/R/F%)		
run0	66.06	78.33	71.67	46.82	57.05	51.43	36.19	44.71	40.00
run1	63.91	85.72	73.22	47.23	65.98	**55.05**	36.88	53.00	**43.50**
run2	59.75	**88.96**	71.48	43.67	**68.79**	53.42	33.68	**54.77**	41.71
run3	64.52	86.19	73.79	47.28	65.40	54.88	36.68	51.87	42.97
run4	**66.18**	84.63	**74.27**	**48.26**	63.27	54.76	**37.76**	50.37	43.16
Median	77.93	69.65	73.56	51.64	54.79	53.17	41.61	39.88	40.73
Average	69.22	68.6	67.03	49.01	48.39	47.74	36.15	35.73	35.22

Results are presented in terms of F1 score. The best scores are highlighted in bold.

Following up, Table 8 compares our best performing result achieved during the challenge with our postchallenge enhancements, namely, the addition of the dynamic negative sampling. Despite our expectations, dynamic sampling did not enhance the novelty score on the test set as it did during the validation phase. Nevertheless, the postchallenge model demonstrated improved performance in terms of entity pair scores (75% versus 73.22%), supporting the idea that the dynamic sampling effectively focuses training on more challenging examples, thereby improving the model’s ability to identify correct pairs. Yet, this improvement in entity pair scoring did not translate into a higher novelty score on the test set. As equally interesting, another observation arises from comparing precision and recall scores. With dynamic sampling, our model achieves more balanced scores, often associated with peak performance in terms of F1 score. This suggests that while dynamic sampling enhances certain aspects of our model’s performance, its impact on the novelty score requires further investigation.

**Table 8.** Comparison between our best challenge run and our postchallenge enhancements.

Configuration	Entity Pair (P/R/F%)			+ Relation (P/R/F%)			+ Novel (P/R/F%)		
Our best submission	63.91	85.72	73.22	47.23	65.98	55.05	36.88	53.00	43.50
Extractor (+ Dynamic Sampling)	73.99	76.04	75.00	54.01	55.82	54.90	42.47	44.01	43.23
Competition best	–	–	**77.07**	–	–	**58.88**	–	–	**44.55**

Results are presented in terms of F1 score. The best scores are highlighted in bold.

Still in Table 8, we also included the performance metrics of the highest-scoring system from the challenge, which surpasses our results by a margin of 1.05 points. Considering this narrow difference, we are optimistic that minor adjustments or the use of a robust ensemble of runs could elevate our system to a comparable level of performance.

#### End-to-end

Lastly, we present, in Table 9, our results regarding our end-to-end system. Similar to the validation results, our end-to-end system underperformed with respect to PubTator 3, showing that possibly our linking results are subpar. It is important to note that we are unable to directly assess the NER and Linking scores; and therefore, we utilize the Relation Extraction score as an indirect measure of the performance of our preceding modules. Moreover, in comparison to the top-performing entry in the competition, our system, when using PubTator 3 as a baseline, demonstrates competitive proximity, trailing by a narrow margin of 1.23 points.

**Table 9.** Comparison between our end-to-end model and PubTator 3 + our Extractor against the competition best.

Configuration	Entity Pair (P/R/F%)			+ Relation (P/R/F%)			+ Novel (P/R/F%)		
Competition best	–	–	55.84	–	–	43.03	–	–	32.75
PubTator 3 + Extractor (BioNExt)	56.64	53.11	54.82	42.27	39.65	40.91	32.56	30.55	31.52
BioNExt (end-to-end)	45.89	40.63	43.10	34.56	30.60	32.46	26.18	23.18	24.59

Below, we present the computation required to operate our end-to-end system. First, in Table 10, we show the total storage needed for all the knowledge bases and the corresponding embedding representations on disk. Then, in Table 11, we show the approximate training and inference times. More specifically, it takes ≈1.72 seconds on average to process a single document, provided that all necessary models and embeddings are loaded into memory.

**Table 10.** Sizes of the raw text entries that we used to perform knowledge base lookup and the corresponding embedding sizes.

Knowledge base	Raw size	Embedding size
NCBI Gene^*a*^ (10)	3.9 GB	5.5 GB
CTD diseases (16, 17)	6 MB	376 MB
MeSH (42)	46 MB	2.6 GB
dbSNP^*b*^ (64)	–	–
NCBI Taxonomy (63)	317 MB	16 GB
Cellosaurus (5)	6.3 MB	595 MB
Total	4.28 GB	25 GB

*a* We only embedded the genes for most frequent species.

*b* As mentioned, we use LitVar2 for performing lookups on dbSNP

**Table 11.** Training and inference times; inference is done over the test set containing 10,000 documents.

		Inference on the test set	
Module	Train	Seconds/Doc	Total
Tagger	00:30:00	0.048	00:08:00
Linker	-	0.6	01:40:00
Extractor	08:00:00	1.08	03:00:00
Total	08:30:00	1.728	04:48:00

## Error analysis

A significant source of inaccuracies within our end-to-end relation and novelty detection model stems from the cumulative effect of errors throughout its various components. Specifically, the success of relation extraction hinges on the accurate identification and linking of entities. If an entity goes unrecognized, it inevitably precludes the possibility of accurately predicting a relation involving that entity. This domino effect of errors offers insight into the significant variances observed between the performances of our integrated end-to-end system and the standalone relation extraction model. Even when we consider the “Extractor” model on its own, we can see the same cascading effect of errors happing when comparing the entity pair, relation and novelty scores, Tables 7 and 8, further harming the final novelty score.

Particularly, the “Linker” module stands out as a primary contributor to these errors by falling short of our expectations. To gain a deeper understanding of where it might have faltered, we devote the remainder of this section to a detailed examination of the most prevalent errors introduced by the “Linker” module.

One error we identified pertains to the dynamic nature of knowledge bases, which are subject to continuous updates and revisions. Through our analysis, we encountered a discrepancy between the codes found in the BioRED dataset and those in our current version of the knowledge bases. This discrepancy arises because certain codes are absent from our knowledge bases; they may have been updated to newer versions, merged with other codes or deprecated. Our versions of the knowledge bases were from February/March 2024, while the ones used in the original BioRED dataset were from before 2022. Table 12 contains the number of unique codes that are present on the validation set of the BioRED dataset that we do not have access. As an example, we are missing the species code 11103. However, upon lookup, we can see that the following code seems to be updated to 3052230. Furthermore, we also verify that PubTator 3 does not suffer from this problem, and it returns these older codes.

**Table 12.** Number of codes we are unable to predict from the validation set.

Entity	Unpredictable	Total
Gene	3	397
Cell Line	0	21
Chemical	3	173
Disease	0	245
Species	1	11
Total	7	847

Another source of error that we identified stems from the interconnected nature of the entity linking process. Effective linking for certain entities is contingent upon the successful linking of dependent entities. For example, accurately linking genes in a document requires prior identification of the species those genes are associated with. Similarly, linking sequence variants is dependent on identifying the specific gene they reference.

This interdependency introduces two layers of complexity to the error landscape. First, we must ensure the accurate prediction and linking of the prerequisite entities, such as species for genes. Second, we must determine the precise relationship between these entities, such as identifying the specific species a gene pertains to or the exact gene a sequence variant is associated with. As mentioned earlier, our approach employs a straightforward algorithm that deduces the species or gene based on the nearest mention. Nevertheless, we have observed instances where this method proves to be inadequate, indicating the need for a more advanced strategy.

For example, in the validation document “Doc510” (PubMed ID: 26847345), our system accurately identifies two references to mice (code: 10090) within the text, inferring that all gene mentions refer to mice. However, all gene mentions in this document actually refer to human (code: 9606), a species not explicitly mentioned in the text.

Lastly, we identified a recurring error in generating tmVar codes with our zero-shot LLM model. According to the tmVar coding standards, a code should begin with one of the letters c, r, g, p or m, representing DNA, RNA, genome, protein and mitochondrial sequences, respectively. A significant portion of the model’s errors stemmed from incorrectly predicting the initial letter of the code. For example, the mention “203G > A” associated with the gene “BRCA2” was incorrectly predicted as “c$\vert$SUB$\vert$G$\vert$203$\vert$A” instead of the correct “g$\vert$SUB$\vert$G$\vert$203$\vert$A.” We believe that these errors could be mitigated by either enriching the model with additional contextual information or by first determining the appropriate initial letter for the code and then conditioning the code generation on that letter.

Another notable error involved the model incorrectly predicting “c$\vert$SUB$\vert$C$\vert$1188$\vert$” instead of the correct “c$\vert$Allele$\vert$C$\vert$1188.” The guidelines specify that “Allele” should be used instead of “SUB” in such contexts. This particular error could be easily rectified with a simple substitution regex, suggesting a straightforward fix for enhancing accuracy in tmVar code generation.

## Conclusions

In this work, we propose an end-to-end biomedical relation extraction model capable of classifying the novelty of identified relations. This innovative model builds upon our system developed for the BioCreative VIII competition, integrating it into a cascading pipeline alongside NER and linking models.

While we encountered challenges, especially in achieving the linking accuracy of established systems like PubTator, our model demonstrated remarkable achievements. Notably, it reached state-of-the-art performance in NER and maintained competitiveness in relation extraction and novelty detection.

Looking ahead, we identify potential areas for enhancement within our model. Namely, refining the linking component to bridge the performance gap with established systems like PubTator is a valuable direction for future work. Additionally, enhancing the relation extraction capabilities, particularly through advancements in our multihead attention mechanism for creating joint representations, presents a promising avenue for further development.

## Footnotes

1
https://github.com/ieeta-pt/BioNExt

2
https://ftp.ncbi.nih.gov/pub/taxonomy/

3
https://www.ncbi.nlm.nih.gov/research/litvar2-api/variant/autocomplete/?query=query gene

4
https://codalab.lisn.upsaclay.fr/competitions/16 381

5
https://huggingface.co/microsoft/BiomedNLP-BiomedBERT-large-uncased-abstract
